# Recombinant Zoster Vaccine Use to Prevent Recurrent Shingles in an Adolescent Renal Transplant Recipient

**DOI:** 10.7759/cureus.83756

**Published:** 2025-05-08

**Authors:** Niloufar Nikfarjam, Paul Grimm

**Affiliations:** 1 Department of Pediatrics, Stanford University, Stanford, USA

**Keywords:** immunosuppression complications, kidney transplant recipient, pediatric kidney transplant, recombinant zoster vaccine, varicella zoster virus reactivation

## Abstract

Shingles, a reactivation of the varicella virus, is more common in immunosuppressed patients. Children with chronic or end-stage kidney disease may not respond effectively to live varicella vaccination, making it controversial post-transplant. Antiviral treatments for shingles can be nephrotoxic, and reducing immunosuppression to enhance immune responses may risk transplant rejection. Recombinant zoster vaccine (RZV) was initially indicated for immunosuppressed adults over 50. We present a case of an adolescent who, after receiving the live varicella vaccine with an observed IgG seroconversion, developed recurrent shingles post-kidney transplant and varicella seronegative status. Antiviral treatments and reduced immunosuppression contributed to graft loss. Post-second transplant, shingles recurred, leading to off-label RZV administration while fully immunosuppressed. Following RZV, there were no rejection or shingles episodes, and strong IgG seroconversion was achieved. Following RZV treatment, guidelines regarding RZV were updated to include individuals over 19, which may potentially benefit pediatric transplant recipients.

## Introduction

Transplant patients undergo immunosuppression to prevent transplant rejection and extend allograft survival, putting them at an increased risk for potential infections. To optimize both graft and patient outcomes, it is critical that potential transplant recipients receive vaccines against all relevant vaccine-preventable diseases prior to transplant [1]. However, there are potential risks to receiving live vaccines after transplantation, as they may cause severe infections in immunosuppressed individuals [2,3]. 

Patients who are immunosuppressed, such as those who have undergone transplantation, have diminished or absent immunity to varicella zoster virus (VZV), which can lead to reactivation of the dormant virus and result in shingles. Live virus vaccines may cause systemic illness in this population. Shingles is nine times more common in immunosuppressed populations compared to those who are immunocompetent [4,5] due to the reactivation of the dormant varicella virus. A relatively new shingles vaccine, the recombinant zoster vaccine (RZV), or Shingrix®, contains recombinant glycoprotein E with the AS01B adjuvant. This vaccine was initially approved for use in adults aged 50 years and older [6]. RZV replaced the live attenuated Zostavax, which was primarily used in individuals aged 60 and older and not recommended for immunosuppressed populations. 

Here, we present the case of an adolescent transplant recipient who lost his first kidney transplant due to complications of recurrent shingles treated with antivirals and reduced immunosuppression. After recurrent shingles developed following the second kidney transplant, we treated him off-label with RZV without reducing immunosuppression, resulting in an excellent serological and clinical response. This article was previously posted to the Research Square preprint server on December 18, 2024.

## Case presentation

This male with end-stage renal disease (ESRD) from congenital nephrotic syndrome received a living donor renal transplant at age two. Prior to this, he had no history of varicella infection but received one dose of live varicella vaccine, which resulted in a positive IgG seroconversion, indicating baseline immunity. Post-transplant, he experienced hypertension and insulin-dependent diabetes mellitus. At age 16, he developed shingles with varicella virus in the skin lesions. He demonstrated diffuse lesions in different stages, including vesicles and scabs on an erythematous base. His glomerular filtration rate (GFR) had been stable with a creatinine baseline of 1.4 mg/dL (reference range: 0.5-1.2 mg/dL) for two years prior to the shingles episode. During admission, he received IV acyclovir (10 mg/kg), saline bolus, and his immunosuppressive medication: tacrolimus 2 mg twice a day (BID) with trough target level of 3-5 ng/mL, prednisone 5 mg once a day (q.d), and mycophenolate 250 mg BID with trough target level of 2-3 mcg/mL, remained unchanged. His varicella zoster virus (VZV) IgG and IgM titers were negative (reference ranges for VZV IgG: <0.60 negative, >=0.60 to <.90 equivocal, >=0.90 positive; reference ranges for VZV IgM: <=0.90 negative, 0.91 to 1.09 equivocal, >-1.10 positive).

**Figure 1:**
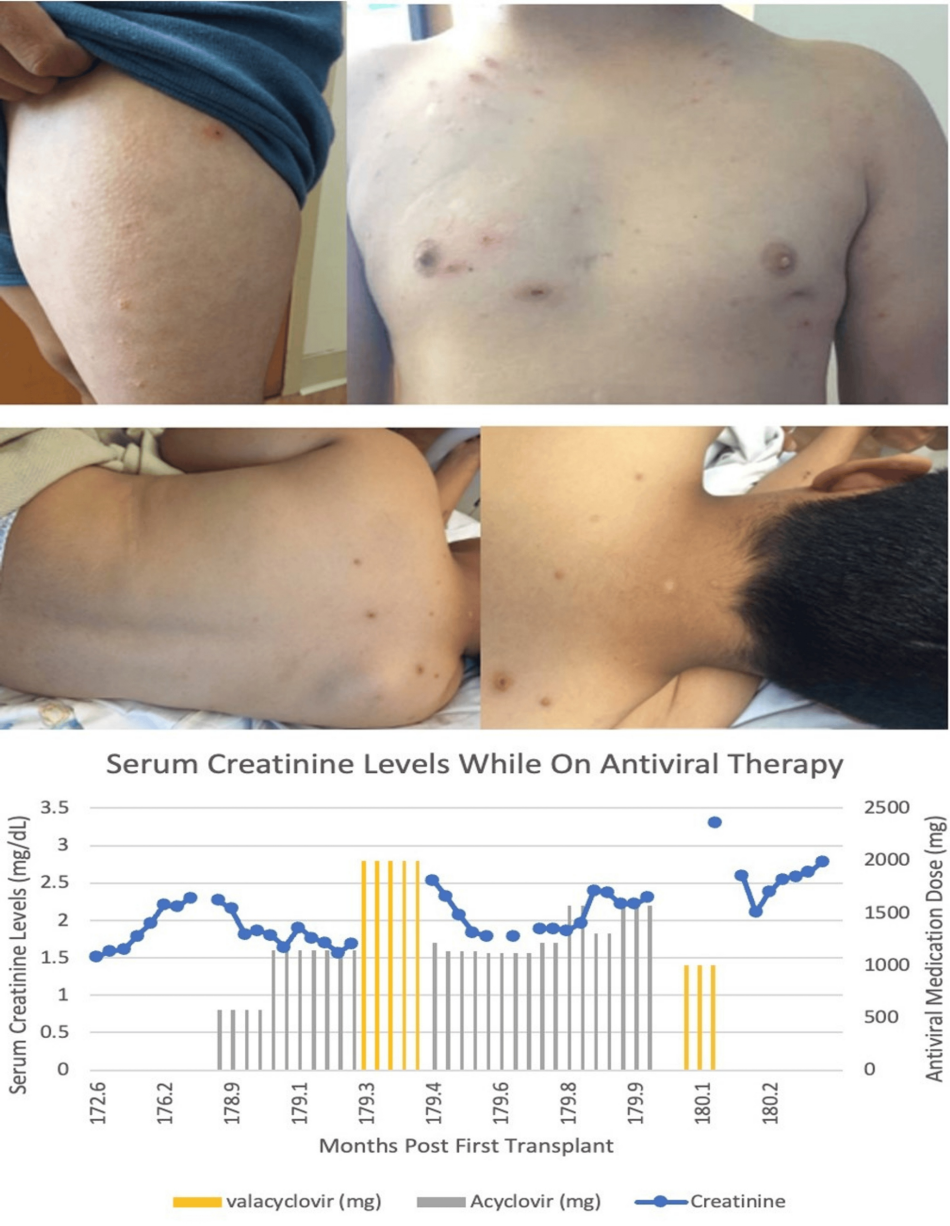
Skin lesions from the first transplant due to shingles and serum creatinine levels in relation to antiviral drug dosing

He was discharged on valacyclovir 1000 mg orally BID for five days but was readmitted one day later with new varicella lesions (Figure 1). On admission, creatinine was elevated at 2.54 mg/dL (reference range: 0.5/1.2 mg/dL). IV Acyclovir was resumed. Mycophenolate was held, and the tacrolimus target was titrated to 3-5 ng/mL. He previously had a low-level donor-specific antibody (DSA) (Cw7 in possible range, mean fluorescence intensity (MFI) 1480). To prevent an amnestic DSA elevation and potential antibody-mediated rejection during reduced immunosuppression, he received 2 g/kg IVIG. New unroofed/crusted lesions developed on his legs, chest, and back, with new papules at previous sites; lesions were VZV-positive by PCR (detected; reference range: not detected). After 16 days in hospital, he was discharged on 1000 mg oral valacyclovir q.d. At discharge, creatinine was 2.31 mg/dL but increased to 3.33 mg/dL after four days (reference range: 0.5-1.2 mg/dL). He was readmitted with acute kidney injury (AKI) from acyclovir toxicity, and creatinine improved to 2.11-2.79 mg/dL (reference range: 0.5-1.2 mg/dL), although it remained elevated from his previous baseline at discharge (Figure 1).

He remained on steroid-based immunosuppression, with tacrolimus 2.5 mg BID and CellCept 500 mg BID, but developed progressive transplant dysfunction so was listed for retransplantation, At 17, he received a preemptive 2/6 antigen-matched deceased donor transplant, with immunosuppression induced by 5 mg/Kg of Thymoglobulin® and prednisone starting at 25 mg q.d., and gradually tapered to 5 mg daily. Post-transplant, he was on tacrolimus with a trough target 5-7 ng/mL and mycophenolate target of 2-4 mcg/mL. Protocol biopsies at six months, one year, and two years showed no rejections and negative DSA testing. Creatinine stabilized at 0.9 mg/dL (reference range: 0.5-1.2 mg/dL). At age 18, he developed <0.5 cm lesions on his lower back and shoulders, appearing vesicular or ulcerated. Excoriations were on his chest and abdomen with 10-20 < 0.5 cm lesions on his buttocks showing vesicular or papular appearance with erythematous bases identical to the shingles he suffered during his first transplant. He was prescribed mupirocin ointment and started on acyclovir 400 mg BID, later reduced to 400 mg q.d. due to creatinine elevation from 0.86 to 1.03 mg/dL (reference range: 0.5-1.2 mg/dL). VZV IgG serology was equivocal, and IgM was negative (reference ranges for VZV IgG: <0.60 negative, >=0.60 to <.90 equivocal, >=0.90 positive; reference ranges for VZV IgM: <=0.90 negative, 0.91 to 1.09 equivocal, >-1.10 positive). He developed new DSA (IgG Class I A29 (MFI 533), Class II DR7 (MFI 772)) in the "possible" range. While on 450 mg q.d. acyclovir, he developed faint erythematous papules on his left shoulder. Acyclovir prophylaxis was continued for a year but stopped due to suspected AKI.

After discussions, the parent consented to the patient receiving RZV at age 20. At the time of vaccination, there were no known active lesions. Tacrolimus trough was 4.4 ng/mL (1.5 mg AM, 2 mg PM), mycophenolate mofetil trough was 1 mcg/mL (500 mg AM,750 mg PM), prednisone was 5 mg q.d. No antiviral agents were administered. There were no vaccine side effects, subsequent monitoring showed no graft dysfunction or indication for biopsy. DSA at five months post-vaccination remained negative. Four years post-vaccination, kidney function remains stable without rejection. VZV IgG is strongly positive at 2285 AU/mL (reference range: <135.00 negative, 135.00-164.99 equivocal, and >=165.00 positive), without prior serotherapy. Creatinine was 0.9 mg/dL (reference range: 0.5-1.2 mg/dL) with an estimated GFR of 112 mL/min/1.73 m^2^. No clinically diagnosed zoster occurred post-RZV. He had a single full-body rash episode that resolved spontaneously within days without healthcare evaluation; the cause is unknown. For a summary of key clinical indicators and the sequence of events, please refer to Table 1. 

**Table 1:** Sequence of events and clinical presentation IV: intravenous; AKI: acute kidney injury; RZV: recombinant zoster vaccine; VZV: varicella zoster virus

Age	Event
Prior to age 2	Varicella vaccine, immunity established
Age 2	Transplant
Age 16	Shingles and vesicular lesions. Treated with IV acyclovir
Age 17	AKI due to acyclovir toxicity. Retransplant
Age 18-20	New lesions
Age 20	RZV Vaccination
Age 24	Follow-up: VZV IgG positive; no proven VZV lesions after RZV vaccine administered

## Discussion

This report highlights the complexities of managing recurrent shingles in an adolescent transplant recipient, especially in the context of immunosuppression. Our findings align with a recent systematic review, which shows that RZV reduces the incidence of herpes zoster by 81% in immunocompromised individuals compared to placebo [7].

In this case, we present an adolescent transplant patient who had recurrent shingles and graft loss, linked to antiviral exposure and reduced immunosuppression. Post-second transplant, he developed multiple shingles and was suspected of AKI. Live varicella vaccine is considered for transplant recipients on minimal immunosuppression, especially those not on antimetabolites; however, our patient received Thymoglobulin® induction and was on an antimetabolite, placing him at risk for live virus vaccination. He developed a new DSA in the "possible" range, making live varicella vaccine more problematic. Given his situation, alternative therapy was needed. After full informed consent, RZV was administered without reducing immunosuppression. There were no further shingles episodes or complications, and follow-up DSA testing showed no spike post-vaccination.

A study reported that mycophenolate mofetil trough levels <2.5 mg/L increased SARS-CoV-2 vaccine seroconversion by sevenfold [8]. Our patient’s trough level was 1 mcg/mL during RZV administration, with documented seroconversion, which may have facilitated seroconversion after the single vaccine dose.

After RZV, our patient had no shingles events for four years. Studies in adult kidney transplant recipients showed increased IgG titers, with 59% exhibiting T cell response after RZV [9]. Another trial showed RZV induces cell-mediated and humoral immune responses when administered 4-18 months post-transplant, with higher responses in the 18-49 age cohort [10]. Stable creatinine levels and no clinical rejection or de novo DSA in this individual case suggest that RZV does not impact allograft function. 

Given the Advisory Committee on Immunization Practices (ACIP)'s recommendation for RZV in immunosuppressed adults [6], our case supports the consideration of RZV for pediatric transplant recipients as well. This long-term follow-up emphasizes the RZV’s potential benefits, even in patients receiving full post-transplant immunosuppression.

## Conclusions

While clinicians are reluctant to use live vaccines due to concerns about potential sequelae, current literature and our case suggest that RZV may represent a valuable option for dealing with VZV disease in pediatric transplant recipients. This case highlights the need for a reevaluation of vaccination strategies in this vulnerable population. Acknowledging potential confounding factors is essential, as patient characteristics and varying levels of immunosuppression may influence the vaccine response. Future studies are essential to assess the safety and efficacy of RZV specifically in pediatric transplant recipients. In 2021, the ACIP recommended RZV for immunosuppressed adults over 19 years. So, vaccinating him today would not be “off-label.” This long-term follow-up shows the vaccine’s potential for the pediatric population despite full post-kidney transplant immunosuppression. Further research is needed to examine the role of RZV in pediatric and young adult transplant recipients.
